# OVERFRAG: An overlapping DNA fragments generator for molecular cloning and synthetic biology

**DOI:** 10.1016/j.dib.2019.103806

**Published:** 2019-03-08

**Authors:** Jose Ribamar Ferreira-Junior, Luciano A. Digiampietri

**Affiliations:** School of Arts, Sciences and Humanities, University of Sao Paulo, R. Arlindo Bettio, 1000, ZIP 03828-000, São Paulo, SP, Brazil

**Keywords:** Gap repair, DNA fragments assembly, Homologous recombination, Synthetic biology, Web tool

## Abstract

DNA synthesis and homologous recombination can be used to simplify molecular cloning and to make synthetic biology easily accessible (M.J. Czar et al., 2009). However, the design of overlapping DNA fragments to construct large molecules is time-consuming and requires verification of several parameters to ensure that fragment synthesis is attainable, given the restrictions found in chemical synthesis of DNA. OVERFRAG is a web-based tool that generates overlapping DNA fragments to assemble either in yeast cells by Gap Repair (H. Ma et al., 1987) or *in vitro* by (D.G. Gibson et al., 2009) and In-Fusion (B. Zhu et al., 2007) methods. The fragments generated are suitable for chemical synthesis and molecular assembly. Some possible uses include cDNA cloning, design of chimeric antibodies and synthetic biology applications. Web tool is freely available at http://www.each.usp.br/digiampietri/overfrag.

**Table:** Specifications table [Please fill in right-hand column of the table below. Each section is mandatory]

Subject area	*Molecular Biology, Bioinformatics*
More specific subject area	*DNA fragments assembly*
Type of data	*Figure and source code*
How data was acquired	*Perl software*
Data format	*Perl language*
Experimental factors	*–*
Experimental features	*–*
Data source location	*Sao Paulo, Brazil, School of Arts, Sciences and Humanities, University of Sao Paulo*
Data accessibility	Data are in this article. Web tool is freely available at http://www.each.usp.br/digiampietri/overfrag
Related research article	*Direct submission to* Data in Brief. *Most relevant research article:* *Gibson, D.G., Benders, G.A., Andrews-Pfannkoch, C., Denisova, E.A., Baden-Tillson, H., Zaveri, J., Stockwell, T.B., Brownley, A., Thomas, D.W., Algire, M.A., Merryman, C., Young, L., Noskov, V*.*N., Glass, J.I., Venter, J.C., Hutchison, C.A., 3rd and Smith, H.O. (2008) Complete chemical synthesis, assembly, and cloning of a Mycoplasma genitalium genome, Science, 319, 1215–1220.*

**Table:** 

**Value of the data** • OVERFRAG will be helpful for designing and cloning of synthetic overlapping DNA molecule fragments, but also may help in the assembly of molecules of both synthetic and natural sources, obtained by PCR amplification. The DNA fragments can be further assembled into yeast cells, likewise by Gibson or In-fusion methods. • Some possible applications of OVERFRAG include gene cloning, cDNA cloning (to assemble exons), chromosome assembly, design of chimeric antibodies, and synthetic biology applications to construct genes, genetic pathways, and genomes. • In synthetic biology, double-stranded DNA molecules with overlapping base pairs allow multiple fragments assembly into yeast to generate larger gene constructs, and remarkably even entire bacterial chromosomes can be assembled [5]. These fragments can be designed using the web tool described here.

## 1 Data

**Fig. 1:**
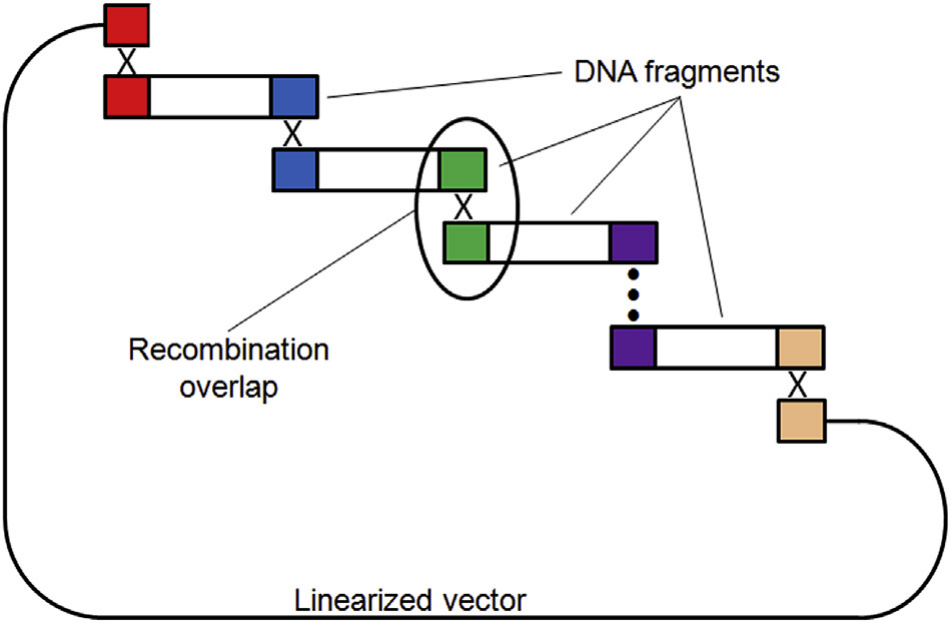
Cloning by homologous recombination. Overlapping DNA fragments and linearized vector are assembled *in vivo* in yeast cells (Gap Repair) or *in vitro* by Gibson or In-Fusion methods. Colored boxes indicate overlap regions. The vertical dotted line between the purple boxes indicates the possibility of more fragments in the assembly.

OVERFRAG is a web-based tool that generates overlapping DNA fragments to assemble [1] either in yeast cells by Gap Repair [2] or in vitro by Gibson [3] and In-Fusion [4] methods. OVERFRAG source code can be found in the supplementary data of this article. The software accepts as input data the promoter, terminator, and coding sequence of a gene and returns DNA fragments, at a chosen length, that overlap a specified number of base pairs. Fig. 1 shows the method of DNA assembly by homologous recombination that can be performed *in vitro* by Gibson and In-Fusion methods, and, also, *in vivo* inside yeast cells by Gap Repair.

## 2 Experimental design, materials, and methods

The tool was developed using Perl programming language (www.perl.org) and the CGI (Common Gateway Interface) library (http://perldoc.perl.org/CGI.html). It receives as input the coding sequence (without introns), the promoter sequence, the terminator sequence, maximum fragment size, overlap size, and the maximum number of extra fragments allowed. The goals of the tool are: concatenate the promoter, coding and terminator sequences, split this concatenation in fragments not bigger than the maximum fragment size, and produce some warnings to inform the user about potential problems of the input sequences or the fragments produced.

To concatenate the three input sequences, and to split the result sequence in overlap regions is, a priori, simple. The challenge lies in keeping the GC-content of each fragment generated between 35% and 65%. This is a restriction imposed by most of the companies which sell synthetic DNA fragments in order to ensure that these fragments can be synthesized. The problem here is the exponential number of different combinations of fragments that can be produced from the input sequences. Moreover, for each fragment, it is necessary to verify the GC-content.

The verification of the GC-content can be efficiently calculated using a dynamic programming strategy: an array of integers is created with the size of the concatenated sequence and each position of the array is filled with the value of accumulated cytosine and guanine nucleotides from the beginning of the sequence until the current position. The GC-content of any subsequence that starts in the position x and finishes at position y from the original sequence is calculated as the difference from the values of the position y and the value of the position x-1 of this array, divided by the size of this subsequence (i.e., y-x+1). Once, the calculation of the GC-content is no longer a problem, the difficult task is to evaluate a significant number of combinations of fragments size in a few seconds.

One traditional solution should be the use of a backtracking algorithm which evaluates all the possible combinations given the restrictions of the maximum fragment size and the overlap size, but, since this approach could take hours to solve even small problems, a simplification was made. Instead of trying all valid combinations of fragment sizes, the backtracking algorithm starts evaluating if the first part of the sequence produces a valid fragment (given the restrictions mentioned before) with the average size (all fragments with the same size). If it produces a valid fragment the algorithm evaluates, recursively, the next fragment. Whenever a fragment is considered invalid, the algorithm tries to resize the current fragment, increasing or decreasing its size up to 25 bp (the limitation of resizing up to 25 bp is being used to ensure that the execution of the tool will not take several seconds). This process is executed considering the production of the minimum number of fragments. Whenever it is not possible to satisfy the restrictions with this minimum value, the system tries to solve the problem with an additional fragment (up to the value of the maximum extra fragments, given by the user as one input of the tool). If no solution is found, the system generates the most basic solution: with the minimum number of fragments with the same size and produces a warning message to inform that this solution does not satisfy one or more restrictions. In this case, the user can use this solution or re-execute the tool changing some parameters, such as maximum number of extra fragments or overlap size.

Some other conditions are also evaluated by the tool, in order to identify if the input sequences satisfy restrictions imposed by companies which sell synthetic DNA fragments. These restrictions are: a DNA sequence must not have homopolymeric runs larger than (a) five cytosine or guanine nucleotides; (b) or nine adenine or thymine nucleotides; and (c) must not contain GAC trinucleotide repeats.

Since the input sequences can have problems (i.e., they may not satisfy the restrictions), the tool only identifies and presents these problems to the user which will choose to keep the sequence or modify it accordingly. It is important to note that there is no automatic solution to this problem since it would imply the modification of the input sequence. Thus, the tool can only produce warnings about these potential problems.
