# Supplementary material for: OVERFRAG: An overlapping DNA fragments generator for molecular cloning and synthetic biology
Source: Data Brief. 2019 Mar 8;23:103806. doi: 10.1016/j.dib.2019.103806 (PMC6660583; doi:10.1016/j.dib.2019.103806)
Supplement: Supplementary file 1 — Multimedia component 1 [file mmc1.docx]

**Source Code**

#########################################################################

#### overfrag.cgi ###

#########################################################################

#!/usr/bin/perl

use CGI;

use strict;

### Global Variables used in Backtracking

my @DIFS;

my @GC_CONTENT;

my $FRAGMENTS;

my $PROBLEM_SOLVED = 0;

my @DIVISIONS;

my @fragments;

my $ADDITIONAL_FRAGMENTS = 3;

### End of Line Characteres

my $c10 = chr 10;

my $c13 = chr 13;

my $meuCGI = new CGI;

print $meuCGI->header(-charset=>"UTF-8");

print $meuCGI->start_html(-title=>"OVERFRAG: overlapping fragments generator tool");

print "<h2>DNA Fragments Generator</h2>\n";

print $meuCGI->start_multipart_form;

my $sequence = "";

my $promoter = "";

my $terminator = "";

my $overlapSize = 50;

my $maxFragmentSize = 500;

if (!defined($meuCGI->param('Clear'))){

if (defined($meuCGI->param('Submit'))){

if (defined($meuCGI->param('sequence'))){

$sequence = $meuCGI->param('sequence');

}

if (defined($meuCGI->param('promoter'))){

$promoter = $meuCGI->param('promoter');

}

if (defined($meuCGI->param('terminator'))){

$terminator = $meuCGI->param('terminator');

}

if (defined($meuCGI->param('maxSize'))){

$maxFragmentSize = $meuCGI->param('maxSize');

}

if (defined($meuCGI->param('overlapSize'))){

$overlapSize = $meuCGI->param('overlapSize');

}

if (defined($meuCGI->param('additional'))){

$ADDITIONAL_FRAGMENTS = $meuCGI->param('additional');

}

}

}

print "Coding Sequence Without Introns:<br><textarea name=\"sequence\" rows=\"10\" cols=\"100\">$sequence</textarea>\n";

print "\n<br>Promoter Sequence:<br><textarea name=\"promoter\" rows=\"4\" cols=\"100\">$promoter</textarea>\n";

print "\n<br>Terminator Sequence:<br><textarea name=\"terminator\" rows=\"4\" cols=\"100\">$terminator</textarea>\n";

print "\n<br>Maximum Fragment Size: <input type=\"text\" name=\"maxSize\" value=\"$maxFragmentSize\" size=\"10\" maxlength=\"20\" />\n";

print "\n<br>Overlap Size: <input type=\"text\" name=\"overlapSize\" value=\"$overlapSize\" size=\"10\" maxlength=\"20\" />\n";

print "<br>Maximum extra fragments: <select name=\"additional\">";

for (my $op=0;$op<=10;$op++){

if ($op==$ADDITIONAL_FRAGMENTS){

print "<option value=\"$op\" selected=\"selected\">$op</option>";

}else{

print "<option value=\"$op\">$op</option>";

}

}

print "</select>\n";

print "\n<br>", $meuCGI->submit(-name=>'Submit', -value => 'Submit');

print " ", $meuCGI->submit(-name=>'Clear', -value => 'Clear');

if (!defined($meuCGI->param('Submit'))){

print "<br><hr>\n";

print $meuCGI->end_form;

print $meuCGI->end_html;

exit(0);

}

if ($overlapSize < 5){

error("ERROR: the overlap size must be greater or equal to 5");

}

if ($maxFragmentSize < 20){

error("ERROR: the maximum fragment size must be greater or equal to 20");

}

if ($maxFragmentSize < $overlapSize + 5){

error("ERROR: the maximum fragment size must be greater or equal to overlap size plus 5");

}

if ($sequence =~/^>/){

if ($sequence =~s/[\n$c10$c13](.+)//){

$sequence = $1;

}

}

$sequence =~s / //g;

$sequence =~s /\n//g;

$promoter =~s / //g;

$promoter =~s /\n//g;

$terminator =~s / //g;

$terminator =~s /\n//g;

$sequence =~s /$c10//g;

$sequence =~s /$c13//g;

$promoter =~s /$c10//g;

$promoter =~s /$c13//g;

$terminator =~s /$c10//g;

$terminator =~s /$c13//g;

my $originalSequence = $sequence;

my $promoterSize = length $promoter;

my $terminatorSize = length $terminator;

my $sequenceSize = length $sequence;

my $numberOfFragments = int (1+($promoterSize + $terminatorSize - $overlapSize + $sequenceSize-1)/($maxFragmentSize-$overlapSize));

$sequence = "$promoter$sequence$terminator";

my $originalNumber = $numberOfFragments;

my $problem = basicFragmentation($sequence, $numberOfFragments);

if ($problem>0){

$DIVISIONS[$numberOfFragments] = length $sequence;

my $tSeq = uc $sequence;

my $len = length $tSeq;

my $prev = 0;

my $char;

for (my $i=0;$i<$len;$i++){

$char = substr $tSeq, $i, 1;

if ($char =~/[GC]/){

$prev += 1;

}

$GC_CONTENT[$i]=$prev;

}

$FRAGMENTS = $numberOfFragments;

rearrange($sequence,0);

my $add = 1;

while ($PROBLEM_SOLVED == 0 && $add <= $ADDITIONAL_FRAGMENTS){

### Trying to solve the problem by using an additional fragment

$numberOfFragments++;

$FRAGMENTS = $numberOfFragments;

$problem = basicFragmentation($sequence, $numberOfFragments);

if ($problem>0){

$DIVISIONS[$numberOfFragments] = length $sequence;

rearrange($sequence,0);

}

$add++;

}

if ($problem > 0 && $PROBLEM_SOLVED==0){

### Unable to solve the problems

$numberOfFragments = $originalNumber;

basicFragmentation($sequence, $numberOfFragments);

}

}

print "<h2>Fragments (g-blocks format)</h2>";

print "<table border=1>\n";

print "<tr><td>Name</td><td>Sequence</td><td>Notes</td></tr>\n";

my $text = "";

for (my $i=0;$i<$numberOfFragments;$i++){

my $p1 = $i+1;

my $len = length $fragments[$i];

print "<tr><td><pre>Fragment$p1\[$len\]</pre></td><td width=300><pre>$fragments[$i]</pre></td><td><pre>Note$p1</pre></td></tr>\n";

}

print "</table><br><br><hr>\n";

print "<h3>Sequence Information: Promoter + Coding Sequence + Terminator</h3>";

my $seq = uc $sequence;

my $tag = "GC";

my $gc5 = 0;

if ($seq =~s/([$tag]{5}([$tag])+)/<font color=#FF0000>$1<\/font>/g){

$gc5 = 1;

}

$tag = "AT";

my $at9 = 0;

if ($seq =~s/([$tag]{9}([$tag])+)/<font color=#00FF00>$1<\/font>/g){

$at9 = 1;

}

$tag = "GAC";

my $gac2 = 0;

if ($seq =~s/(($tag){1}($tag)+)/<font color=#0000FF>$1<\/font>/g){

$gac2 = 1;

}

my $seqToPrint = filter($seq);

print "<pre>$seqToPrint</pre>\n";

if ($gc5+$at9+$gac2>0){

print "<b>Warnings:<\/b>\n<pre>";

if ($gc5==1){ print "<font color=\#FF0000>Too many GCs.</font><br>"; }

if ($at9==1){ print "<font color=\#00FF00>Too many ATs.</font><br>"; }

if ($gac2==1){ print "<font color=#0000FF>Too many GACs.</font><br>"; }

print "</pre>\n";

}

content(uc $sequence);

print "<br><hr>\n";

print "<h3>Fragments Information<\/h3>";

print "<table border=1>\n";

print "<tr><td>Name</td><td>Length</td><td>GC-content</td></tr>\n";

my $gc_problem = 0;

for (my $i=0;$i<$numberOfFragments;$i++){

my $p1 = $i+1;

my $len = length $fragments[$i];

my $gc = gc($fragments[$i]);

my $tagGC = "";

if ($gc<35 || $gc>65) {

$gc_problem = 1;

$tagGC = "<font color=#00FFFF>*</font>";

}

print "<tr><td><pre>Fragment$p1</pre></td><td><center><pre>$len</pre></td><td><center><pre>$gc\%$tagGC</pre></td></tr>\n";

}

print "</table>\n";

if ($gc_problem==1){

print "<font size=-1 color=#00FFFF>Many systems recommend GC-content between 35% and 65%.</font>\n";

}

print "<br><hr>\n";

print $meuCGI->end_form;

print $meuCGI->end_html;

exit(0);

sub error{

my $message = shift;

print "<font color='#FF0000'><h2>$message</h2></font>";

print $meuCGI->end_form;

print $meuCGI->end_html;

exit 0;

}

sub gc{

my $seq = shift;

$seq =~s/ //g;

my $total = length $seq;

$seq =~s/[GCgc]//g;

my $remain = length $seq;

my $gc = 10000*($total-$remain)/$total;

$gc = int ($gc+0.499999);

return $gc/100;

}

sub filter{

my $seq = shift;

my $bases = 80;

my $output = "";

my $len = length $seq;

my $htmlTag = 0;

my $contBases = 0;

my $char;

for (my $i=1;$i<=$len;$i++){

$char = substr $seq, $i, 1;

if ($htmlTag){

if ($char eq ">") { $htmlTag = 0; }

}else{

if ($char eq "<") { $htmlTag = 1; }

else { $contBases++; }

}

if ($contBases == $bases){

$output = "$output$char<br>";

$contBases = 0;

}else{ $output = "$output$char"; }

}

return $output;

}

sub content{

my $seqTemp = shift;

my $len = length $seqTemp;

my $remaining = $len;

my $char;

my $content = 0;

print "<table border=1>\n";

print "<tr><td>Nucleotide</td><td>Content</td></tr>\n";

while ($seqTemp ne ""){

$char = substr $seqTemp, 0, 1;

if (length $seqTemp > 0 && $seqTemp =~s/$char//g){

$content = $remaining - length $seqTemp;

if ($content > 0){

$remaining = length $seqTemp;

$content = 10000*$content/$len;

$content = int ($content+0.499999);

$content = $content / 100;

print "<tr><td><center><pre>$char</td><td><center><pre>$content\%</td></tr>\n";

}else{ $seqTemp = ""; }

}else{ $seqTemp = ""; }

}

print "</table>\n";

}

sub rearrange{

my $seq = shift;

my $frags = shift;

$frags++;

my $tGC;

my $v = 0;

my $gc0 = 0;

my $ind0 = 0;

my $ind1 = 0;

my $fragLen;

my $len = length $sequence;

if ($frags<$FRAGMENTS) {

if ($frags>1){

$ind0 = $DIVISIONS[$frags-2]+$DIFS[$frags-2]+1-$overlapSize;

$gc0 = $GC_CONTENT[$ind0-1];

}

for (my $j=0;$j<=50;$j++){

if ($j>0){

$v = int (($j+1)/2);

if ($j%2==0) { $v = -1*$v; }

}

$ind1 = $DIVISIONS[$frags-1]+$v;

$fragLen = $ind1-$ind0+1;

if ($fragLen > $overlapSize && $fragLen <= $maxFragmentSize){

$tGC = ($GC_CONTENT[$ind1]-$gc0)/($fragLen);

if ($tGC >= 0.35 && $tGC <= 0.65){

$DIFS[$frags-1] = $v;

$DIFS[$frags] = 0;

rearrange($seq,$frags);

}

}

if ($PROBLEM_SOLVED) {return;}

}

}else{

$ind0 = $DIVISIONS[$frags-2] + $DIFS[$frags-2] + 1 - $overlapSize;

$gc0 = $GC_CONTENT[$ind0-1];

$ind1 = -1 + length $seq;

$fragLen = $ind1-$ind0+1;

if ($fragLen > $overlapSize && $fragLen <= $maxFragmentSize){

$tGC = ($GC_CONTENT[$ind1]-$gc0)/($ind1-$ind0+1);

if ($tGC >= 0.35 && $tGC <= 0.65){

$PROBLEM_SOLVED = 1;

my $start = 0;

my $end;

for (my $w=0;$w<$FRAGMENTS;$w++){

$end = $DIVISIONS[$w] + $DIFS[$w];

$fragments[$w] = substr $sequence, $start, $end-$start+1;;

$start = $end - $overlapSize+1;

}

}

}

return;

}

}

sub basicFragmentation{

my $sequence = shift;

my $numberOfFrags = shift;

my $totalSize = ($numberOfFrags-1)*$overlapSize + $promoterSize + $terminatorSize + $sequenceSize;

my $basesPerFragment = int ($totalSize/$numberOfFrags);

my $extraBases = $totalSize % $numberOfFrags;

my $problem = 0;

if ($numberOfFrags>1){

my $plusOne = 0;

my $initialPosition = 0;

my $finalPosition = 0;

for (my $i=0;$i<=$numberOfFrags-1;$i++){

if ($extraBases>0) {

$plusOne = 1;

$extraBases--;

}else{

$plusOne = 0;

}

$finalPosition = $initialPosition + $basesPerFragment + $plusOne;

$DIVISIONS[$i] = $finalPosition;

$fragments[$i] = substr $sequence, $initialPosition, $basesPerFragment + $plusOne;

$initialPosition = $finalPosition-$overlapSize;

my $tGC = gc($fragments[$i]);

if ($tGC < 35 || $tGC>65){

$problem++;

}

}

}else{

$fragments[0] = "$promoter$sequence$terminator";

my $tGC = gc($fragments[0]);

if ($tGC < 35 || $tGC>65){ $problem = 1; }

else { $problem = 0; }

}

if ($problem == 0) { $PROBLEM_SOLVED = 1; }

return $problem;

}

**Acknowledgements**

This research did not receive any specific grant from funding agencies in the public, commercial, or not-for-profit sectors.

**References**

[1] M.J. Czar, J.C. Anderson, J.S. Bader, and J. Peccoud, Gene synthesis demystified, Trends Biotechnol. 27 (2009) 63-72. https://doi.org/10.1016/j.tibtech.2008.10.007.

[2] H. Ma, S. Kunes, P.J. Schatz and D. Botstein, Plasmid construction by homologous recombination in yeast, Gene 58 (1987) 201-216. https://doi.org/10.1016/0378-1119(87)90376-3.

[3] D.G. Gibson, L. Young, R.Y. Chuang, J.C. Venter, C.A. Hutchison, 3rd and H.O. Smith, Enzymatic assembly of DNA molecules up to several hundred kilobases, Nat. Methods 6 (2009) 343-345. https://doi.org/10.1038/nmeth.1318.

[4] B. Zhu, G. Cai, E.O. Hall and G.J. Freeman, In-fusion assembly: seamless engineering of multidomain fusion proteins, modular vectors, and mutations, Biotechniques 43 (2007) 354-359. https://doi.org/10.2144/000112536.

[5] D.G. Gibson, G.A. Benders, C. Andrews-Pfannkoch, E.A. Denisova, H. Baden-Tillson, J. Zaveri, T.B. Stockwell, A. Brownley , D.W. Thomas, M.A. Algire, C. Merryman, L. Young, V.N. Noskov, J.I. Glass, J.C. Venter, C.A. Hutchison, 3rd and H.O. Smith, Complete chemical synthesis, assembly, and cloning of a Mycoplasma genitalium genome, Science 319 (2008) 1215-1220.

**Figure Caption**

Fig. 1. Cloning by homologous recombination. Overlapping DNA fragments and linearized vector are assembled *in vivo* in yeast cells (Gap Repair) or *in vitro* by Gibson or In-Fusion methods. Colored boxes indicate overlap regions. The vertical dotted line between the purple boxes indicates the possibility of more fragments in the assembly.
